# Assessing Heavy Metal and PCB Exposure from Tap Water by Measuring Levels in Plasma from Sporadic Breast Cancer Patients, a Pilot Study

**DOI:** 10.3390/ijerph121215013

**Published:** 2015-12-09

**Authors:** Anne Marie Zimeri, Sara Wagner Robb, Sayed M. Hassan, Rupali R. Hire, Melissa B. Davis

**Affiliations:** 1 Department of Environmental Health Sciences, University of Georgia, Athens, GA 30602, USA; zimeri@uga.edu; 2 Department of Epidemiology & Biostatistics, University of Georgia, Athens, GA 30602, USA; swagner@uga.edu; 3 Laboratory for Environmental Analysis, University of Georgia, Athens, GA 30602, USA; shassan@uga.edu; 4 Department of Genetics, Medical Partnership, University of Georgia, Athens, GA 30602, USA; Davidson Life Sciences Building, 120 East Green St., Athens, GA 30602, USA; rupahire@uga.edu

**Keywords:** sporadic breast cancer, PCBs, heavy metals, uranium

## Abstract

Breast cancer (BrCA) is the most common cancer affecting women around the world. However, it does not arise from the same causative agent among all women. Genetic markers have been associated with heritable or familial breast cancers, which may or may not be confounded by environmental factors, whereas sporadic breast cancer cases are more likely attributable to environmental exposures. Approximately 85% of women diagnosed with BrCA have no family history of the disease. Given this overwhelming bias, more plausible etiologic mechanisms should be investigated to accurately assess a woman’s risk of acquiring breast cancer. It is known that breast cancer risk is highly influenced by exogenous environmental cues altering cancer genes either by genotoxic mechanisms (DNA mutations) or otherwise. Risk assessment should comprehensively incorporate exposures to exogenous factors that are linked to a woman’s individual susceptibility. However, the exact role that some environmental agents (EA) play in tumor formation and/or cancer gene regulation is unclear. In this pilot project, we begin a multi-disciplinary approach to investigate the intersection of environmental exposures, cancer gene response, and BrCA risk. Here, we present data that show environmental exposure to heavy metals and PCBs in drinking water, heavy metal presence in plasma of nine patients with sporadic BrCA, and Toxic Release Inventory and geological data for a metal of concern, uranium, in Northeast Georgia.

## 1. Introduction

Although breast cancer (BrCA) has been the most commonly diagnosed cancer among women in the United States for several decades (one out of eight women will be diagnosed during their lifetime), we are still unraveling its highly varied etiology, which is dependent upon the interactions of exogenous exposures with endogenous genetic predispositions. A notable body of work associates mutations of specific genes with an increased incidence of familial BrCA syndromes. However, 85% of BrCA cases in the United States each year are sporadic, suggesting that the causes also arise from a non-heritable source [1]. One potential important mechanism in sporadic BrCA is environmental exposures. For example, we are aware of the relationship between estrogenic responses to BrCAs and increased BrCA risks [2,3]. Environmental exposure(s) may be estrogenic, which interact with genetic backgrounds and initiate responses that increase the likelihood of BrCA occurrence.

Other associations for sporadic BrCA include factors such as dietary patterns, obesity, occupational exposure, stress levels, smoking and alcohol consumption, all of which are related to both the natural and the built environment [4,5,6,7,8]. These factors can be assessed with patient interviews. In addition, several other methods exist to assess environmental exposures; including, proximity and exposure to toxic releases that can be tracked using the US EPA’s toxic release inventory (TRI), analysis of toxicants in drinking water (analyzed with historical data compared to laboratory data), and/or human biometric data (*i.e.*, the analysis of patient plasma). Certain classes of environmental exposures, such as those related to chemical agents or toxins and energy transmission, have been repeatedly implicated in genotoxic mechanisms for lung cancer and leukemias [9]. Therefore, it stands to reason that these agents may also be involved with development of other cancers, such as BrCA. Several recent environmental investigations have defined environmental agents associated with BrCA incidence [10,11,12,13,14,15,16,17,18]; however, some of these agents have not been shown to be mutagenic or teratogenic, therefore it is unclear exactly how these agents drive the mechanisms of tumorigenesis. In addition, environmental agents that cause sporadic BrCA incidence may have reversible effects, as is evident in intervention studies that indicate removing the harmful environmental agent reduces cancer risk [19,20].

For this pilot study, we selected heavy metals and PCBs as potential environmental factors that contribute to sporadic BrCA because they have been shown to persist at high levels within our geographic region, have been implicated in epigenetic modifications, and are linked to cancer etiology. For example, uranium has been associated with several types of cancer, including breast. Moreover, an examination of naturally-occurring uranium in the adjacent state of South Carolina, which has similar geochemical properties, identified an increased risk of certain cancers in areas with elevated ground water uranium and more groundwater consumption (Wagner, 2010). The mechanism by which these cancers arise is not fully clear, however, it is thought that heavy metals may interfere with immune responses and bio-electromagnetic fields inside the body [21], may cause inflammation, or may act by driving the methylation of DNA, which can lead to epigenetic effects including sporadic cancers.

PCBs, which we hypothesize may play a role in causing sporadic BrCAs, can be ingested in drinking water when these components are present. There are more than 200 known PCBs, however the most common eight PCBs are small (mono- through octo-). Exposure to PCBs in laboratory animals has caused liver cancer [22]. Testing for these toxicants in drinking water and comparing them with sporadic BrCA cases will allow for a correlation analysis that may provide the basis for additional testing. For example, if elevated levels of PCBs are found in drinking water in BrCA cases that present as sporadic, additional genetic testing can be performed to determine whether or not methylation has occurred in the DNA which may lead to the cancer itself and to epigenetic effects in future generations.

Here, we present a pilot study aimed at correlating heavy metals, especially uranium, and PCBs in drinking water, and in patient plasma samples, with predicted concentration generated from spatial analysis of environmental data from the Toxic Release Inventory in North Georgia of nine women who present sporadic breast cancer. We found several instances of high heavy metal concentrations in plasma samples despite all water samples testing below EPA limit. We also found PCB levels (for mono-octo-) above EPA limits in almost all of the patients.

The overall goal of this study was to characterize environmental exposures, using pre-existing geographic environmental data, water samples, and human plasma samples in sporadic breast cancer cases. We hypothesized that sporadic breast cancer (BrCA) susceptibility, as indicated by the epigenetic regulation of cancer genes, is related to an individuals’ sensitivity to exposure to specific environmental agents. While many individuals are exposed to shared or similar environments and environmental agents (EA), some members of the population may have greater sensitivity to these agents, translating into an increase in cancer susceptibility. This increase in cancer risk may be regulated by epigenetic mechanisms controlling the expression response of cancer genes involved in tumor etiology.

Risk assessment should comprehensively incorporate exposures to exogenous factors that are linked to a woman’s individual susceptibility. However, the exact role that some EAs play in tumor formation and/or cancer gene regulation is unclear. In this pilot project, we begin to use a multi-disciplinary approach to investigate the intersection of environmental exposures, cancer gene response and breast cancer risk.

## 2. Materials and Methods

Toxic release inventory data (TRI): TRI data are publicly available through the US EPA website, and can be easily accessed. Data include the locations of hazardous sites, the amount of on-site releases, and the types of chemicals released. To determine whether there was a correlation between our breast cancer patients and toxic chemical releases, we generated maps to determine how far the patients lived from known toxic releases. We then collected data on how far each patient was from a toxic release inventory, and on which toxins were released at that time. Patient participants signed an informed consent document, and protocols and surveys were approved by the University of Georgia Internal Review Board.

Materials: All standard and QA/QC solutions were obtained from reputable sources (PE EXPRESS, the Perkin Elmer Corporation, Waltham, MA, USA; SPEX CertiPrep, Metuchen, NJ, USA; PlasmaCAL, Melville, NY, USA). Trace metal grade nitric acid was purchased from Fisher Scientific, USA. Pure argon gas (99.999%) was used for ICP-MS.

Sample preparation: 0.2 mL aliquots of plasma were transferred to 120 mL teflon digestion vessels, followed by the addition of 5 mL of nitric acid. After setting aside for 30 min, the capped vessels were placed in a microwave oven for 20 min at power level 5, then for another 20 min at power level 4. The digested samples was reconstituted by addition of 50 mL of double deionized water and kept for ICP/MS analysis. Analysis of metals in plasma was preceded by microwave digestion with concentrated nitric acid to destroy the organic part and mineralize the sample. The multi-element calibration standard is provided as 10 mg/L in 5% nitric acid and is not in plasma matrix.

ICP-MS analysis: The Elan 9000 (Perkin Elmer-Sciex) inductively coupled plasma mass spectrometer (ICP/MS) equipped with an AS 91 auto-sampler (PerkinElmer, Norwalk, CT, USA) was used for metal analysis.

The instrument conditions used were: FR power—1100 W, nebulizer gas flow (L/min)—0.90. Measuring conditions: scan mode—peak hopping, replicate time—1 s, dwell time—50 ms, sweeps—40, integration time—2000 ms, replicates—3. The sensitivity (detection limit) for metals under this method is 0.1 to 0.5 ng/L. The linearity was up to 100 ppm, with a relative standard deviation less than 2%. Standards were obtained from Perkin Elmer, Inc. (Branford, CT, USA) (Table 1).

Calibration: Three-point multi-element calibration curves were used for standardization.

Water sampling method: Tap water samples from each of the breast cancer patients’ kitchen faucets were collected. The tap water was run on the cold setting for five minutes prior to collection in an amber glass 1 L bottle that was previously autoclaved. Bottles were kept on ice until the analysis could be performed. Metal concentrations were determined using inductively couple plasma mass spectroscopy, similar to that mentioned for plasma samples in the above section.

PCB analysis: The aim of this study was to get the concentration of total PCBs within each group of congeners based on the number of chlorine atoms in each group. A GC/MS/MS method using the selected ion storage technique of ion trap mass spectroscopy was used, in which 8 segments represented PCBs from the monochloro up to the octochloro derivatives. The response area for the detected congeners (within each chloroderivative) were summed to give the total concentration reported in Table 2. The water samples (about 1000 mL) were passed through conditioned C18 cartridges at a rate not exceeding 5 mL/ min. The loaded cartridges were extracted by methanol first, then with dichloromethane at a flow rate not exceeding 0.5 mL/ min. The collected extracts were vortex shaken, then concentrated by evaporation under nitrogen almost to dryness, treated with 1 mL methanol and evaporated again to dryness, then dissolved in 1 ml methanol. The methanol extracts were analyzed for PCB content using the procedure published in the application note 00993 by VARIAN. The sensitivity (detection limit) for PCBs under this method is 0.05 to 0.15ng/L. The linearity was up to 10 mg/L with a relative standard deviation less than 4%. Authentic PCBs were obtained as 100 ug/mL mixture containing from monochlorobiphenyl to octachloro derivative from Absolute Standards, Inc. (Hamden, CT, USA).

Plasma Preparation: Briefly, 200 to 300 microliters (0.2–0.4 mg) was mixed with 10.0 mL of methanol and 5.0 mL MTBE (methyl-t-butyl ether):hexane (1:1) followed by sonication for 15 min, It was Centrifuged at 1600 rpm for 15 min and the supernatant was mixed with 5.0 mL of MTBE—hexane 1:1 in a separate tube. The residue was again mixed with MTBE—hexane and sonicated and the procedure was repeated four times until no more residue was left, ensuring the maximum digestion. The supernatant after each round of digestion and centrifugation was pooled for each sample and concentrated to 1.0 mL by passing Nitrogen at 55 °C. This concentrated sample was passed through column of activated florisil (activated 16H at 130 °C, used 1.8 g per sample) equilibrated with MTBE—hexane 1:1 mix. The sample was eluted through the column with dichloro methane (DCM) and concentrate to dryness by passing Nitrogen at 55 °C. The dried contents were dissolved in 1.0 mL of methanol and analyzed with GLC.

Uranium mapping and predicted exposures: The National Uranium Resource Evaluation (NURE) Hydrogeochemical and Stream Sediment Reconnaissance (HSSR) Program, which is maintained by the United States Energy Research and Development Administration, explored uranium resources in addition to more than 40 chemical constituents [23]. Systematic sampling of stream sediments, soils, groundwater, and surface water throughout the US began in 1975 and ended in 1983-84 through for Department of Energy labs. Several labs were designated a specific geographic area that maintained their own sample collection and used various analytic techniques, making the data accuracy dependent of its lab, the study, analytical methodology, the analyte, and its concentration.

Inverse distance weighting (IDW) and ordinary kriging are the most widely used spatial interpolation methods for environmental data. IDW considers that the measured value closest to the prediction location has more influence on the predicted value than those further away. A variety of neighborhoods, directionality influences, and weighting functions were evaluated in order to optimize the prediction result. Kriging predicts similarly to inverse distance weighting expect that the prediction weights are generated using a semivariogram model, which determines model fit. Use of a semivariogram also enables anisotropic aonsideration. IDW was chosen as the optimal methods for uranium PLUTO sediment and NURE water data.

A GIS (ArcMAP software, version 10.0; ESRI, Redlands, CA, USA) was used to evaluate the environmental data sources and develop exposure surfaces for each EA of interest.

**Table 1:** Linearity, Relative Standard Deviation (RSD), and Limits of Detection (LOD).

Element	Linearity	RSD	LOD
	R2	%	ng/L
Ag	0.9998	0.5	0.2
Al	0.9999	1.8	0.5
As	0.9975	0.8	0.3
Br	0.9996	0.6	0.4
Cd	0.9999	0.4	0.1
Fe	0.9994	1.8	0.5
Pb	0.9999	0.2	0.1
U	0.9999	0.2	0.1

**Table 2:** Results of the analysis of selected metals (mg/L) in plasma from 9 patients with sporadic BrCA.

Metal in mg/L	EEG-0001	EEG-0002	EEG-0003	EEG-0004	EEG-0005	EEG-0006	EEG-0007	EEG-0008	EEG-0009
Ag	0.018	0.004	0.002	0.591	0.002	0.004	0.002	0.001	0.009
Al	0.470	0.000	0.000	0.000	0.000	3.278	0.000	0.000	0.000
As	0.024	0.010	0.002	0.011	0.002	0.006	0.003	0.006	0.019
Br	44.398	17.869	13.875	9.748	6.270	7.860	12.971	23.084	25.950
Cd	0.001	0.000	0.000	0.011	0.000	0.000	0.000	0.000	0.000
Fe	5.684	10.789	5.344	4.797	2.631	6.349	3.905	4.748	24.082
Pb	0.024	0.017	0.051	0.023	0.072	0.031	0.009	0.153	0.062
U	0.000	0.001	0.000	0.000	0.000	0.000	0.000	0.000	0.000

## 3. Results

This pilot study analyzed the potential exposure risk to heavy metals, and eight PCBs among nine breast cancer patients. Each of the water samples taken from the kitchen taps in the patients’ homes were examined for the concentration of 66 metals, including uranium. Each sample had metals present, but all were below the EPA maximum control limit (MCL) (data not shown). However, several patients had high metal levels in their plasma samples (Table 2).

Though the source of these metals may have been the tap water, accumulation in the plasma could be related to other factors or conditions specific to those patients. In addition, treatments, supplements, and occupational exposures may have played a role in these concentrations. These two possibilities will be analyzed with extensive surveys that were administered to the pilot patients.

Through this study, we found that sporadic breast cancer cases and PCBs were related. We found detectable levels of the PCBs tested to be apparent in both the tap water sampled and the plasma of the nine patients in the pilot study (Table 3 and Table 4).

**Table 3:** PCB content in water (µg/L).

PCB->	Mono-	Di-	Tri-	Tetra-	Penta-	Hexa-	Hepta-	Octo-
Patient (EEG-)								
0001	3.6161	54.5880	0.4028	40.7895	13.7433	0.3046	3.0526	0.0074
0002	17.7455	53.9755	0.9479	443.6842	15.6684	0.4569	4.2105	0.0233
0003	3.9063	37.3441	0.5213	118.9474	13.3690	0.1523	2.7368	0.0203
0005	3.5045	32.3550	0.3081	17.8947	11.9786	0.1523	3.2632	0.0208
0007	2.5223	28.2183	0.2962	99.2105	11.8717	0.2030	3.3684	0.0213
0008	3.0357	32.1102	0.2133	25.2632	11.5508	0.1015	3.1579	0.0146
0009	2.8571	32.0267	0.2133	9.2105	10.8556	0.2030	2.7368	0.0135

**Table 4:** PCB content in plasma (µg/L).

PCB	Mono-	Di-	Tri-	Tetra-	Penta-	Hexa-	Hepta-	Octo-
Patient (EEG-)								
0001	34.6514	45.4449	2.0311	62.6566	94.2195	1.4503	27.0677	0.4260
0002	17.1429	44.4098	1.8957	40.0000	75.7219	0.8122	21.0526	0.1934
0003	29.6875	71.8263	2.4882	36.8421	88.2353	1.0152	30.5263	0.2166
0004	17.6339	31.8207	2.1327	27.6316	47.0588	0.7614	11.5789	0.1000
0005	15.6822	45.0574	1.8836	21.5924	46.8943	0.2603	12.9555	0.1014
0006	16.2815	56.7274	1.8121	37.1517	51.2740	0.5972	14.2415	0.1181
0007	14.0625	51.7187	1.6588	25.0000	45.4545	1.2690	13.6842	0.0992
0008	29.9908	97.8813	6.1976	20.2429	67.4619	2.3428	16.7341	0.1042
0009	31.4335	162.4920	4.4147	18.7455	75.5989	1.6689	17.3035	0.1165

PCBs are known to cause cancer in laboratory animals and are among the EPA’s top 10% of toxic chemicals. Though their manufacture and import was banned in 1979, many of the transformers and capacitors in which they were used are still in use today. Once these are spent, their proper disposal is key in preventing human and environmental exposure. Therefore, it stands to reason that the PCB exposures in these patients may have played a role in the development of sporadic BrCa. The EPA limit for PCBs is 0.5 µg/L based on EPA test method 8082A. Most patients had similar tap water results with only a couple of exceptions. Patient EEG-0002, who uses unfiltered well-water for cooking and bathing, but drinks bottled water, had higher levels for mono-, di-, tri-, tetra, and hexa-chloro PCB compounds. This may be explained by a TRI releases in her area, or possibly fish consumption. PCBs are stored and bioaccumulate in the fatty tissues of animals including humans. Patient EEG-0001 had a high di-PCB level, which was similar to the level observed in patient EEG-0002. This patient drinks filtered well-water. Large volumes of PCBs have been introduced to the environment through the burning of PCB-containing products, vaporization from PCB-containing coatings and materials, releases into sewers and streams, improper disposal of PCB-containing equipment in non-secure landfill sites and municipal disposal facilities, and by other routes (such as ocean dumping). These water levels of PCBs, however, do not directly correlate with the high PCB levels we found in the study patients’ plasma. High plasma levels may, instead, be attributable to differential accumulation, based on physiology or other common exposures aside from water. For example, PCB exposure can result from consuming fish, fatty meats, and dairy.

**Figure 1:**
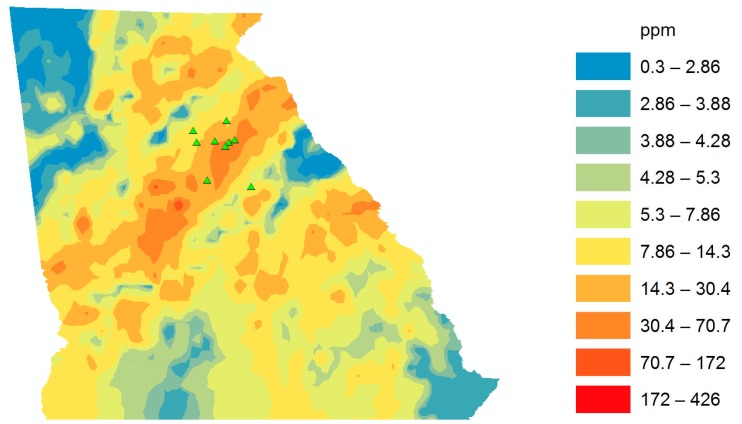
GIS map of Uranium in GA. Small green triangles represent the vicinity of patients’ residences in North Georgia. Predicted uranium exposure via sediment for each cancer patient based on GIS data in ppm.

Northeast Georgia has a high, naturally occurring level of uranium in soils and sediment. According to GIS data, uranium levels are 20%–50% higher in the Northeast Georgia regions compared to the remainder of the country (Figure 1).

We wanted to determine whether this uranium would be present in our study patients’ tap water and plasma. In patient EEG-0002’s tap water, 2 µg/L of uranium was detected. All other patients’ water registered below the detectable limits of the following: in water: 5 ng/L, in soils and sediments, based on dry wy; 0.5 µg/kg, in tissues based on wet wt: 5–2 µg/kg. However, patient EEG-0002 did not have a detectable amount of uranium in her plasma. Surprisingly, patient EEG-0001 had 0.001 ppm uranium in her plasma, despite not having a detectable limit in tap water at her residence. The EPA has a maximum contaminant level goal (MCLG) for uranium at zero, however the current MCL allowable is, as of 2003, 30 µg/L.

## 4. Conclusions

We presented data that show environmental exposure to heavy metals and PCBs in drinking water, heavy metal presence in plasma of nine patients with sporadic BrCA, and Toxic Release Inventory and geological data for a metal of concern, uranium, in Northeast Georgia. Certainly, our conclusions are limited by the small sample size, which did not include patients with familial breast cancer nor patients who did not present breast cancer. In addition, we will be able to add more information on patients’ histopathology, tumor stage, and residential history, in order to more accurately assess the environmental exposures at their residences, especially through drinking water. These issues will be resolved in a much larger study that will result from this pilot. The epidemiological observations of our genes’ responses to the environment are evident in cancer incidence rates correlating with environmental exposures (EEs); however, the systematic investigations of gene response dynamics among populations with variable specific exposures are still largely unknown. We will use a pilot case-control study to comprehensively determine first whether specific EAs in our region are linked to BrCA incidences in rural populations, and, second, if these EAs are associated with altered oncogene or tumor suppressor expression regulation. In addition, future studies with more patients will allow us to look for a correlation between EAs and prognosis. The transcriptional expression levels of a gene are highly regulated by the relative stress of its localized chromatin-structure. This epigenetic state for any given gene may vary among cell types, and can be directly influenced by exogenous cues [24,25,26]. This remodeling includes modifications to both DNA and the chromatin proteins that package the DNA. Alterations in the methylome create a certain cellular micro-environment by virtue of inhibiting gene responsiveness. Epigenetic studies are expanding to include the pursuit of environmental interactions that impact BrCA risk by altering gene regulation [27,28,29,30,31]. For instance, the biological impact of Arsenic has been recently reviewed and includes global DNA methylation changes in lung cancer. In ER-negative BrCA disease similar silencing of the ER locus has been associated with methylation dynamics within patient cohorts [32,33]. We will utilize Next Generation Sequencing (NGS) genomics tools (Targeted Methyl-Sequencing) to systematically investigate the epigenetic state of a targeted set of genes in relation to BrCA [34,35,36,37] incidence and EA exposures that we determined from this study.

While positive family history of the disease can have a clear association with the onset of malignancy, as having a first-degree relative with a positive diagnosis doubles the risk of BrCA, and two such relatives triple the risk; the cases with association to mutation are rare. In familial clusters of cancer, investigations of risk factors indicate that the true causative factors go beyond the scope of inherited genetic traits. Not only do family groups share genetic makeups, but also lifestyle factors and EAs. These shared exposures may lead to shared DNA methylation pattern alterations that disrupt tumor suppressor type gene function and can lead to tumor growth. First, however, exposures must be correlated with sporadic breast cancer cases to determine their efficacy in promoting cancer.

The EPA reports and tracks more than 600 chemicals in its TRI. Though we tested for eight PCBs that are reported, there are several other chemicals included that may be risk factors for BrCA, as well as hundreds of others that may act in combination to pose a risk for BrCA. It is possible that some of the chemicals may even mask the effects of others; therefore, there may be other factors that contributed to the BrCA cases in this study that are unknown. Further examinations of patient residential and occupational history may serve to narrow the field of contributors to their disease.

This pilot study serves as the first level of an approach to identify environmental factors that may work to cause epigenetic effects that lead to the presentation sporadic BrCA. Following this work, a larger cohort study that will include methyl-seq DNA analysis will be used to determine with more certainty the environmental factors that can lead to sporadic BrCA.
